# Telehealth in arts therapies for neurodevelopmental and neurological disorders: a scoping review

**DOI:** 10.3389/fpsyg.2024.1484726

**Published:** 2024-12-18

**Authors:** Ērika Reitere, Jana Duhovska, Vicky Karkou, Kristīne Mārtinsone

**Affiliations:** ^1^Department of Health Psychology and Paedagogy, Rīga Stradiņš University, Riga, Latvia; ^2^Research Centre for Arts and Wellbeing, Edge Hill University, Ormskirk, United Kingdom

**Keywords:** art therapy, arts therapies, dance movement therapy, expressive arts therapy, music therapy, neurodevelopmental disorders, neurological disorders, telehealth

## Abstract

**Background:**

Arts therapies, encompassing art therapy, music therapy, drama therapy, and dance movement therapy with the broader practice of expressive arts therapies, have demonstrated positive outcomes in the treatment of neurodevelopmental and neurological disorders (NNDs). Integrating arts therapies into telehealth has become increasingly important to improve accessibility for people with mobility impairments or those living in remote areas. This study aims to map the existing body of literature to provide an in-depth overview of telehealth in arts therapies for individuals with NNDs.

**Methodology:**

This scoping review followed the PRISMA guidelines. Six databases were systematically searched, with 2,888 articles screened for eligibility. Inclusion criteria focused on primary research peer-reviewed articles in English that addressed telehealth arts therapies for NNDs.

**Results:**

Seventeen telehealth studies published between 2009 and March 2024 were included, with a notable increase in publications after 2020. The studies covered various neurodevelopmental disorders, including autism spectrum disorders, attention deficit hyperactivity disorder (ADHD), Rett syndrome, and neurological disorders such as stroke, epilepsy, cerebral palsy, central nervous system (CNS) tumors, dementia, Alzheimer’s disease, Parkinson’s disease, spinal cord injuries, and mild cognitive impairment. Music therapy was the most widely studied modality. Interventions ranged from therapeutic singing and songwriting to virtual reality experiences. Different platforms and specialized virtual environments were used alongside pre-recorded sessions. Positive benefits included psychological enrichment, social connectivity, cognitive improvements, and brain changes, although some studies reported mixed or no significant effects in certain areas.

**Conclusion:**

Telehealth in arts therapies significantly benefits individuals with NNDs, improving accessibility and providing psychological, emotional, social, and cognitive benefits. The positive benefits observed highlight the potential of these interventions to improve overall well-being and daily functioning. Future research may focus on high-quality qualitative studies and neuroimaging assessments to further validate the impact of telehealth arts therapies.

## Introduction

### Arts therapies

Arts therapies are forms of psychotherapy that aim to improve the physical and mental health of patients or clients by addressing social issues, preventive work, and personal growth. These therapies integrate artistic tools and creative processes with verbal reflection for both assessment and intervention in settings that may be individual, group-based, in-person, or remote, under the guidance of certified professionals^1^ (Mārtinsone and Duhovska, 2023). Karkou and Sanderson (2006) define the field as: “… the creative use of the artistic media as vehicles for non-verbal and/or symbolic communication, within a holding environment, encouraged by a well-defined client-therapist relationship, in order to achieve personal and/or social therapeutic goals appropriate for the individual” (p. 46). Internationally, the field includes various creative therapeutic practices with different naming conventions. In some countries, one profession encompasses multiple specializations like art, music, drama, and dance movement therapy, while others recognize these as separate professions (Mārtinsone and Duhovska, 2023). Expressive arts therapy further combines these modalities for enhanced therapeutic impact (Malchiodi, 2020). Arts therapists, equipped with advanced graduate-level training, practice in various settings, including hospitals, schools, community centers, correctional facilities, hospices, and private practices (Shafir et al., 2020). Telehealth also broadens access, increasing the reach of arts therapies and extending their therapeutic potential to remote and underserved areas.

### Neural mechanisms and brain networks in arts therapies

In arts therapies, several brain networks work together to support healing through aesthetic experiences. The sensory-motor network, or “embodied brain,” facilitates physical engagement with art forms such as dance or music, allowing patients to express and reflect emotions through movement, linking these actions to personal memories, and enhancing the therapeutic process. Rather than passively receiving stimuli, this network actively constructs perceptual experiences using interoceptive (internal) and exteroceptive (external) cues, grounding mental experiences in physical sensations and promoting a holistic therapeutic engagement (Vaisvaser, 2021; Vaisvaser et al., 2024).

The default mode network, or “predictive brain,” plays a role in anticipating and interpreting sensory input based on past experiences. Predictive processing, which involves a dynamic flow of expectations (top-down) and sensory data (bottom-up), minimizes prediction errors by either adjusting actions or updating internal models. Engaging in unpredictable, creative tasks within creative arts therapies helps clients reshape mental models, promoting adaptive responses to new experiences and expanding their sense of self (Vaisvaser, 2021). The salience network draws attention to emotionally impactful moments in therapy, helping individuals to focus on significant experiences, while the reward network reinforces engagement and curiosity by making aesthetic experiences pleasurable, thus enhancing emotional investment and motivation (Vaisvaser et al., 2024).

The relational brain also plays an essential role, involving mechanisms such as mental simulation and synchronization– neural processes that support empathy, understanding, and shared emotional experiences through the mirror neuron system (Vaisvaser, 2021). Finally, the executive control network supports cognitive control, enabling patients to balance introspection with actionable problem-solving. Together, these integrated network activities allow creative arts therapies to promote resilience and well-being, combining cognitive, emotional, and physical healing into a unified therapeutic experience (Vaisvaser et al., 2024).

### Telehealth and its growing importance in arts therapies

Various terms have been used to describe remote healthcare services, including telemedicine, telerehabilitation, teleintervention, and telehealth. Among these, telehealth has emerged as a comprehensive term that encompasses a wide range of remote healthcare activities and services (Clements-Cortés et al., 2023). Telehealth is a broad term that describes the use of electronic communications to provide clinical services and other types of health information. It is used for a wide range of activities aimed at general health and well-being. Telehealth provides an efficient means of connecting patients with healthcare providers in situations where in-person visits are not possible. The most used approach in telehealth includes real-time interactions between a patient and a healthcare provider through video conferencing, telephone calls, live chat, or non-simultaneous communication via online platforms or mobile applications (American Telemedicine Association, 2020). In recent years, virtual reality has also gained increasing popularity as an innovative tool in telehealth, offering immersive experiences that can enhance patient engagement and therapeutic benefits (Lian, 2023).

Telehealth gained particular prominence during the COVID-19 pandemic when the need for social distancing and the burden on healthcare facilities underscored the importance of remote healthcare services (Smith et al., 2020). The use of telehealth services experienced an unprecedented increase during the pandemic and is expected to remain at high levels going forward compared to pre-pandemic times, indicating a likely long-term change in the field (Pierce et al., 2021). Since 2020, the growth of telehealth has also seen a notable increase in the use of arts therapies within telehealth services (Feniger-Schaal et al., 2022). This period highlighted the potential of telehealth to improve access, efficiency, and safety in healthcare, with its benefits contributing to a significant acceleration in its adoption and integration into mainstream healthcare practices (Doraiswamy et al., 2020). Furthermore, a meta-analysis by Lin et al. (2022) found that there were no substantial differences in treatment outcomes between teletherapy and in-person therapy, suggesting that teletherapy can be an equally effective alternative to traditional, in-person approaches, thus supporting its continued integration into healthcare practices.

Telehealth in arts therapies is essential for several reasons. First, it greatly expands access to therapeutic services for individuals with limitations that prevent them from attending traditional in-person therapy sessions and provides opportunities for ongoing engagement and monitoring that are critical to maintaining therapeutic gains. This is particularly important for individuals with mobility impairments or who live in remote areas with limited healthcare resources. Furthermore, the flexibility of telehealth platforms allows for more personalized and convenient therapy schedules, which can be critical for patients with complex care needs (Cole et al., 2021), and, ultimately, telehealth is associated with lower costs (Dorsey and Topol, 2016; Egede et al., 2018). Receiving therapy at home can provide a sense of safety for patients who may feel more comfortable in their own environments (Ashwick et al., 2019; Christensen et al., 2020).

### Challenges and creative adaptations in telehealth arts therapies

The integration of telehealth into arts therapies offers substantial benefits but also presents distinct challenges that impact both technological and therapeutic dimensions. It requires significant adjustments on the part of both therapists and clients, affecting various aspects of the therapeutic process (Markowitz et al., 2021). In addition to technological barriers, such as the lack of high-speed internet, especially in remote areas, the telehealth environment also presents inherent legal, ethical, safety, privacy, and confidentiality challenges that can disrupt the therapeutic process (Burger and Wuensch, 2021; Levy et al., 2017; Lin et al., 2021; Weinberg and Rolnick, 2019).

The physical separation inherent in remote sessions can complicate the triangular therapeutic relationship between the client, therapist, and artwork, requiring therapists to put extra effort into creating a safe and engaging digital environment (Zubala et al., 2021). Therapists have reported feeling less effective in performing certain therapeutic skills in teletherapy settings compared to in-person therapy, particularly in relational elements such as empathy, warmth, and support (Lin et al., 2021). Remote sessions may feel more physically and mentally exhausting than in-person sessions as they require greater concentration, restrict physical movement, and limit the therapist’s ability to read the patient’s cues, often leaving the patient feeling tense and confined (Markowitz et al., 2021). The restriction of physical movement can be a limiting factor in remote dance movement therapy, as movement is essential to fostering expression, body awareness, and emotional processing in therapeutic settings.

The lack of tactile qualities in remote tools can limit the sensory experiences that are considered therapeutic in traditional art therapy. This often requires access to specific materials or musical instruments that may not be easily accessible to patients in remote settings, potentially limiting the creative process. To overcome these limitations, therapists may need to adapt by recommending materials that are readily accessible or adaptable at home or by sending them directly to the client’s home (Sharpe and Hinz, 2023). Although remote therapy may initially seem to limit art-based interventions, it can offer opportunities to enhance creativity, which in turn enriches and revitalizes the therapeutic process (Mor, 2022).

Using digital tools in arts therapies can lead to innovative therapeutic approaches that are not possible in traditional settings, thereby enriching the therapeutic experience. The flexibility of the digital medium provides creative freedom, enabling safe experimentation with art and easy modifications through tools such as virtual reality or tablet-based drawing, fostering client self-exploration. Digital artmaking can help reduce client discomfort associated with “messy” materials or direct tactile engagement, making it particularly suitable for individuals with tactile sensitivities or developmental disabilities. Additionally, the digital medium’s ability to document and revisit the creative process helps clients gain insight into their therapeutic progress (Zubala et al., 2021). All telehealth advantages are especially crucial for individuals with NNDs, who often face significant barriers to traditional in-person therapy and can significantly benefit from the expanded accessibility and flexibility that telehealth offers.

### Understanding neurodevelopmental and neurological disorders

Neurodevelopmental disorders are behavioral and cognitive disorders that emerge during early developmental phases and are characterized by substantial challenges in acquiring and performing specific intellectual, motor, language, or social skills (American Psychiatric Association, 2022). Developmental stages generally refer to the period before an individual reaches the age of 18, regardless of when a diagnosis is made (World Health Organization, 2022). The etiology of many neurodevelopmental disorders remains highly complex and not fully understood. It is generally believed that these disorders are primarily caused by genetic or other factors present from birth (Thapar et al., 2017). However, insufficient environmental stimulation or lack of adequate learning opportunities and experiences can also contribute to the development of neurodevelopmental disorders (Johnson et al., 2016; Nelson et al., 2019). The most common neurodevelopmental disorders include, among others, autism spectrum disorder, ADHD, intellectual development disorder, and developmental learning disorder (World Health Organization, 2022).

Neurological disorders are diseases of the central or peripheral nervous system that affect the brain, spinal cord, cranial nerves, peripheral nerves, nerve roots, vegetative nervous system, neuro-muscular junction, and muscles (Da Cunha De Sá-Caputo et al., 2021). These disorders include epilepsy; headache disorders (including migraine); neurodegenerative disorders (including dementia and Parkinson’s disease); cerebrovascular diseases (including stroke); neuroinfectious/neuroimmunological disorders (including meningitis, HIV, neurocysticercosis, cerebral malaria, multiple sclerosis); neuromuscular disorders (including peripheral neuropathy, muscular dystrophies and myasthenia gravis); traumatic brain and spinal cord injuries; and cancers of the nervous system (World Health Organization, 2023).

Although the causes of many neurological disorders remain unknown, genetic, epigenetic, and external factors have been implicated in the onset and progression of these diseases (Kelly et al., 2020). These conditions can lead to significant functional impairment, affecting individuals’ ability to perform daily activities and reducing their quality of life. The increasing incidence of death and disability from neurological disorders is widely recognized as a global public health issue, and this burden is expected to grow in the coming decades due to the aging of the population (Ding et al., 2022). The economic impact of these disorders is also substantial, with costs associated with healthcare, lost productivity, and long-term care. Individuals with neurological disorders often require extensive social and economic support due to their physical, cognitive, and psychosocial challenges (Ransmayr, 2021). Given the extensive needs of individuals with neurological disorders, arts therapies can provide essential support alongside traditional medical care. Research indicates that arts therapies have positive outcomes in the treatment of NNDs (Ettinger et al., 2023; Lo et al., 2019; Raglio, 2015), offering a complementary approach to address the complex physical, cognitive, and psychosocial challenges faced by these individuals.

### Scoping review objectives

With the significant increase in the use of telehealth, it is necessary to examine its current state in the field of art therapies to compile the interventions used, identify the most common delivery tools, and determine the benefits of telehealth for individuals with neurodevelopmental or neurological disorders. This review can provide professionals with insights on how to work with NNDs using arts therapies via telehealth. Therefore, this scoping review aims to map the body of literature to provide an in-depth overview of the current state of arts therapies in the field of telehealth for NNDs.

### Scoping review questions

*RQ1: What* NNDs *are included in published articles on telehealth in arts therapies?*

*RQ2: What therapeutic methods are used in telehealth arts therapies for individuals with* NNDs*?*

*RQ3: What are the prevalent delivery platforms and digital tools used in telehealth interventions of arts therapies for individuals with* NNDs*?*

*RQ4: What are the benefits of telehealth arts therapies for individuals with* NNDs*?*

## Methods

### Scoping review design

As a scoping review, it aimed to map the existing literature on a particular topic by identifying key themes and knowledge gaps. Unlike systematic reviews, scoping reviews do not conduct a quality assessment of the included studies, nor do they produce meta-analyses (Golden et al., 2021). In conducting the review, meticulous methodology and standardization were observed in accordance with the PRISMA Scoping Review guidelines (Page et al., 2021; Tricco et al., 2018) and the six-step methodological framework proposed by Arksey and O'Malley (2005). This approach comprises the following stages: (1) identification of the research question; (2) identification of relevant studies; (3) selection of studies; (4) charting of data; (5) collation, summarization, and reporting of results; (6) consultation (optional) (Arksey and O'Malley, 2005).

### Search strategy

#### Inclusion and exclusion criteria

Each study included in this review met the following inclusion criteria: (a) primary research articles published in peer-reviewed journals, (b) no restriction on publication date, (c) quantitative, qualitative, or mixed methods studies, (d) written in English, (e) a variety of genders, cultural backgrounds, contexts of adversity, and age groups (children, adults, and the elderly), (f) with a neurodevelopmental or neurological disorder (clinically diagnosed or with symptoms identified through assessment tools), (g) the study was explicitly situated in the arts, music, dance movement, drama or expressive arts therapy, (h) provided or supervised by a qualified professional (such as an art therapist, music therapist, dance movement therapist, drama therapist, expressive arts therapist, or instructor), (I) therapy delivered via telehealth^2^ (j) with no limitation on the length or duration of the therapy session, (k) no restriction on the format (group or individual), (l) no restriction on the outcomes assessed.

Studies were excluded for the following reasons: (a) systematic reviews, book chapters, gray literature, (b) studies without full text in English, (c) studies describing only the experiences of therapists or staff without focus on individuals with neurodevelopmental or neurological disorders, (d) studies in which only students were present without the involvement of a certified therapist, or therapeutic intervention was led by a physiotherapist, dance instructor, or nurse, (e) therapy that was not provided in telehealth setting (f) in cases of duplicate data across multiple studies, only the study with the most comprehensive dataset was retained, and the others were excluded to prevent redundancy.

#### Information sources

Six databases were searched in March 2024: PubMed, Scopus, ProQuest, Science Direct, Taylor & Francis, and Web of Science. A second search was performed using Google Scholar, resulting in seven additional papers identified as relevant.

#### Search methods

The search process consisted of several stages: initial search screening, main search screening, and supplemental manual search screening (Page et al., 2021). Two librarians from an academic institution assisted in preparing the search strings for each database and conducting both the initial and primary screening procedures.

The search strategy included the following keywords of relevant articles: “Drama therapy” or “Music Therapy” or “Art Therapy” or “Dance Therapy” or “Creative art therap*” or “Art* psychotherapy” or “Expressive therap*” and various terms used in the existing literature for telehealth, such as “Telemedicine” or “eHealth” or “virtual” or “remote” or “digital” or “emedicine” or “online therapy” or “telehealth.” Boolean operators AND/OR were used to connect keywords; the symbol * was used to abbreviate the word. Search fields included title, abstracts, Medical Subject Headings (MeSH) terms, MESH Major topic and Text Word. See Table 1 for search strings used in each database.

**Table 1:** Search strings for database systems.

Database system	Search strings
PubMed	“drama therapy”[Text Word] OR “Music Therapy”[Text Word] OR “Art Therapy”[Text Word] OR “Dance Therapy”[Text Word] OR (“Music Therapy”[MeSH Major Topic] OR “Art Therapy”[MeSH Major Topic] OR “Creative art therap*”[Text Word] OR “Art* psychotherapy”[Text Word] OR “Expressive therap*”[Text Word] OR “Dance Therapy”[MeSH Major Topic]) AND (“Telemedicine”[Text Word] OR “ehealth”[Text Word] OR “virtual”[Text Word] OR “remote”[Text Word] OR “digital”[Text Word] OR “emedicine”[Text Word] OR “online therapy”[Text Word] OR “Telemedicine”[MeSH Terms])
Scopus	“drama therap*” OR “Music Therap*” OR “Art Therap*” OR “Dance Therap*” OR “Creative art therap*” OR “Art* psychotherapy” OR “Expressive therap*” AND (Telemedicine OR ehealth OR virtual OR remote OR digital OR emedicine OR “online therapy”) Filters: “Article” and “English”
ProQuest	subject((“drama therap*” OR “Music Therap*” OR “Art Therap*” OR “Dance Therap*” OR “Creative art therap*” OR “Art* psychotherapy” OR “Expressive therap*”)) AND subject((Telemedicine OR ehealth OR virtual OR remote OR digital OR emedicine OR “online therapy”)) Filters: APA PsycArticles, APA PsycTherapy, MEDLINE, and Psychology Databases
Science Direct	“drama therapy” OR “Music Therapy” OR “Art Therapy” OR “Dance Therapy” AND (Telemedicine OR virtual OR remote OR digital OR “online therapy”) Filters: “Research article”; “Review article”; “English”
Taylor & Francis	“drama therap*” OR “Music Therap*” OR “Art Therap*” OR “Dance Therap*” OR “Creative art therap*” OR “Art* psychotherapy” OR “Expressive therap*” AND (Telemedicine OR ehealth OR virtual OR remote OR digital OR emedicine OR “online therapy”)
Web of Science	“drama therap*” OR “Music Therap*” OR “Art Therap*” OR “Dance Therap*” OR “Creative art therap*” OR “Art* psychotherapy” OR “Expressive therap*” AND (Telemedicine OR ehealth OR virtual OR remote OR digital OR emedicine OR “online therapy”) Filters: “English”
Google Scholar	“drama therapy” OR “Music Therapy” OR “Art Therapy” OR “Dance Movement Therapy” AND (Telemedicine OR virtual OR telehealth OR remote OR digital OR “online therapy”)

Neurodevelopmental and neurological disorders were intentionally excluded from the search strategy. Each publication was meticulously evaluated to determine its alignment with NNDs as categorized by the ICD-11: International Classification of Diseases. This search approach was recommended by librarians to avoid excluding relevant studies due to search query length restrictions. Specifically, not all possible disorder terms were included in the search queries to prevent unintentionally omitting relevant studies.

#### Selection of evidence sources

**Figure 1:**
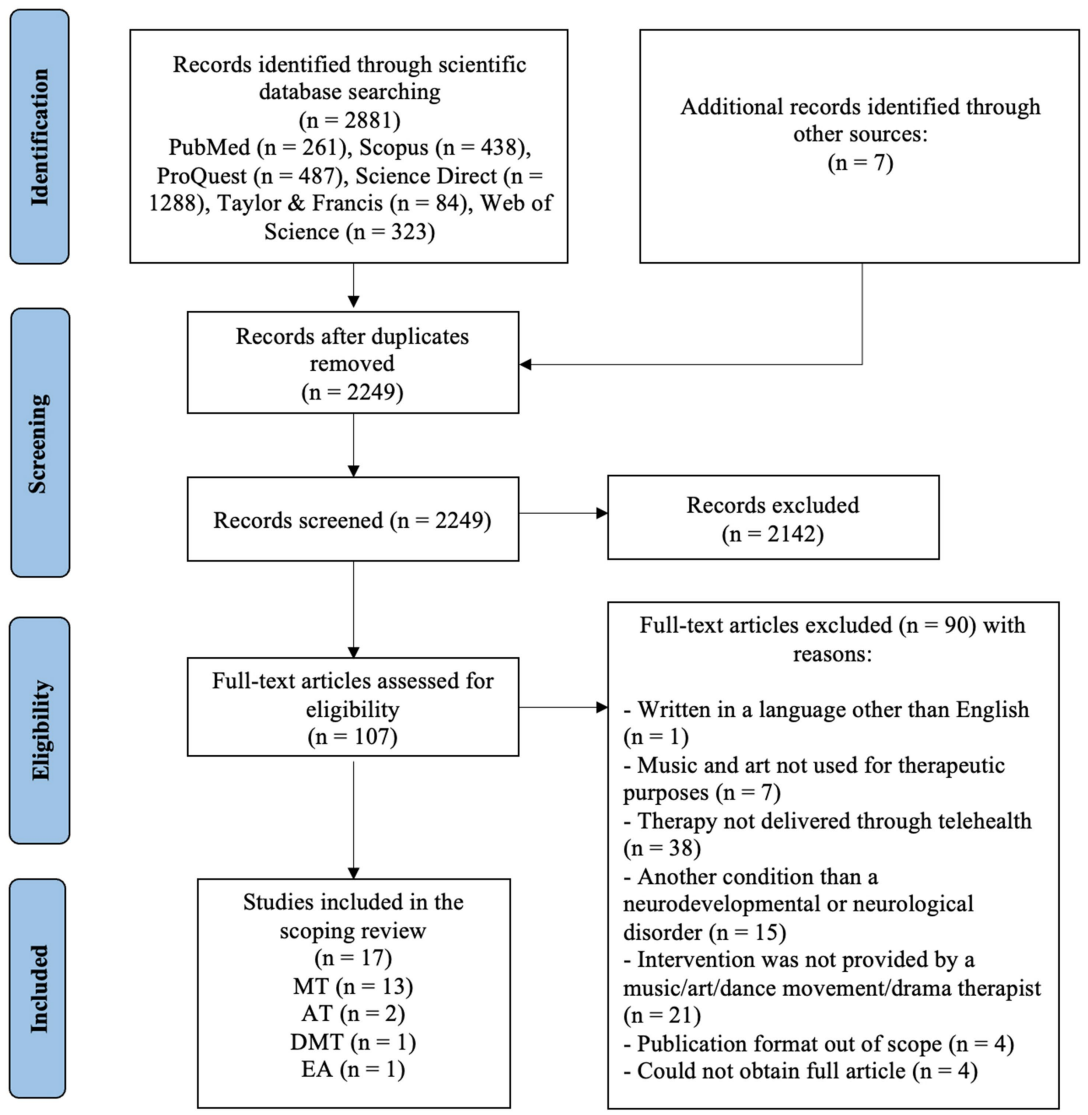
PRISMA flow diagram for scoping review.

All publications were screened for title and abstract. Articles were screened and selected on Rayyan,^3^ a digital platform for systematic article review, and each reviewer was unaware of the selections made by other reviewers. All available sources up to March 2024 were uploaded into Rayyan, where duplicates were identified and removed, resulting in 2249 articles for initial screening. The blind feature in Rayyan was then activated to enable independent abstract-level screening and separate flagging of selected articles. Studies were selected by two independent researchers, who screened the selected articles by title and abstract according to the inclusion/exclusion criteria. After the initial screening, the blind function was removed, allowing the researchers to identify articles with a disagreement between the two reviewers. The researchers reviewed and discussed these articles by re-reading the abstracts until an agreement was reached. A total of 2,142 articles were excluded as irrelevant based on the inclusion criteria, leaving 107 articles for full-text review (Figure 1).

#### Selection of the articles

The first author reviewed all articles in full text and began the initial data analysis and classification stages. After a thorough review of the content, the second researcher performed a secondary selection according to the inclusion and exclusion criteria. Both researchers reviewed and discussed the excluded and included articles until agreement was reached. The full-text articles were classified into three categories: included, excluded, or maybe. After analyzing the content of 107 full-text articles, 90 were excluded because they did not meet the following inclusion/exclusion criteria: (1) written in a language other than English (*n* = 1); (2) music and art were not used for therapeutic purposes (*n* = 7); (3) interventions were not conducted through telehealth (*n* = 38); (4) another condition than a NNDs (*n* = 15); (5) intervention was not provided by a music/art/dance movement/drama therapist (*n* = 21); (7) publication format out of scope (*n* = 4); (6) could not obtain the full article (*n* = 4). In total, 17 articles met all the inclusion criteria for this scoping review.

#### Data charting and analysis

The research team developed a data synthesis table in Microsoft Word 2024. The first and second authors charted the data, analyzed them, and subsequently peer-reviewed the categorization. The following data were obtained: author(s) name (s), publication year, the country where the research was conducted, study design (qualitative, quantitative, or mixed methods), participant information, disorder, session information (time, intensity, duration), intervention (steps, program), delivery platforms and tools, assessment tools, reported results and limitations. A summary of the main findings can be found in Supplementary Table S1.

## Results

### General characteristics

Seventeen studies published from 2009 to 2024 were included in this scoping review. Of these, 14 studies (82.35%) were conducted between 2020 and 2024, indicating a notable increase in research following the COVID-19 pandemic. The geographic distribution of the studies was as follows: United States (*n =* 5); United Kingdom (*n =* 4); Australia (*n =* 3); Italy (*n =* 1); China (*n =* 1); Republic of Korea (*n =* 1); Israel (*n =* 1); Ireland (*n =* 1). The majority of the 17 studies were qualitative studies (*n* = 8), mixed-method (*n* = 6), and a minority were quantitative studies (*n* = 3). The therapeutic focus of the review was predominantly music therapy (*n* = 13), with smaller contributions from art therapy (*n* = 2), expressive arts (*n* = 1), and dance movement therapy (*n* = 1). Sample sizes varied widely across study types: quantitative studies included between 8 to 73 participants, qualitative studies ranged from 1 to 14 participants, and mixed method studies included sample sizes from 1 to 87 participants. Participants ranged in age from 4 years to 87 years, representing a diverse demographic. Treatment lengths varied from single-session interventions to 15-month programs, with session durations ranging from brief 5-min interventions to comprehensive 90-min sessions. The frequency of sessions varied widely as well, ranging from three times a day to once a week.

### NNDs in published articles on telehealth in arts therapies

Articles published on telehealth in arts therapies have addressed various NNDs. Three studies of telehealth music therapy included Parkinson’s disease (Shah-Zamora et al., 2024; Stegemöller et al., 2020; Tamplin et al., 2024), three studies of dementia (Clark et al., 2024; Dassa, 2024; Kelly et al., 2024), and two studies of autism (Devlin, 2022; Quigley and MacDonald, 2024). Additional studies included Alzheimer’s disease (Quail et al., 2021), Asperger’s syndrome (Baker and Krout, 2009), stroke, epilepsy, CNS tumors, and other neurological conditions (Bonakdarpour et al., 2021), spinal cord injury (Tamplin et al., 2019), cerebral palsy, and various genetic syndromes (Bompard et al., 2023). Telehealth art therapy studies covered autism spectrum conditions with various diagnoses (Datlen and Pandolfi, 2020) and ADHD (Kim and Chung, 2024). One published study of expressive arts therapy addressed mild cognitive impairment (Luo et al., 2023), and one in telehealth dance movement therapy focused on autism (Moo and Ho, 2023).

### Therapeutic methods used in telehealth arts therapies for individuals with NNDs

The interventions used in the context of telehealth arts therapies for individuals with NNDs were diverse and tailored to address the specific challenges of each individual. The interventions used in music therapy via telehealth for patients with Parkinson’s disease were delivered in group settings. Shah-Zamora et al. (2024), with 16 adults, conducted sessions in small groups with each patient paired with their caregiver. A common feature across all music therapy studies for Parkinson’s disease was the focus on group singing, breathing support, and vocal exercises (Shah-Zamora et al., 2024; Stegemöller et al., 2020; Tamplin et al., 2024). Sessions also incorporated verbal check-in conversations (Shah-Zamora et al., 2024) and 30-min social interaction practices (Tamplin et al., 2024) to promote engagement and reduce isolation.

Music therapy sessions for people with dementia included the presence of a spouse or care partner, offering both individual (Clark et al., 2024; Dassa, 2024; Kelly et al., 2024) and group (Clark et al., 2024) formats. The Clark et al. (2024) study of nine adults and their care partners used a two-phase approach. The first phase consisted of four individual therapy sessions with a care partner, followed by six group sessions that incorporated an online therapeutic songwriting program that was flexible and sensitive to the needs of the participants. For Alzheimer’s disease, interventions included cognitive stimulation activities, reminiscence therapy, and musical activities such as listening to music, playing an instrument, and singing, as well as physical exercises (Quail et al., 2021).

Music therapists have used telehealth to work with individuals with autism, and therapies have included improvisational experiences such as guitar playing and many play elements to replicate improvisational experiences they experience during the face-to-face work (Devlin, 2022). Quigley and MacDonald (2024) conducted a Rock’n Roll music therapy project delivered by community musicians and music therapists that included improvisation, songwriting, and the process of recording two songs, as well as musical and emotional recall exercises. For individuals with stroke, epilepsy, CNS tumors, and other neurological conditions, individualized sessions incorporated live, improvised violin music with pitches aligned to the human vocal range (131–524 Hz) and tailored to participants’ musical preferences (Bonakdarpour et al., 2021). Music therapy for adult patients with spinal cord injuries involves group singing online and in virtual reality via the app (Tamplin et al., 2019). Interventions for children with genetic syndromes included parent-home music therapy using the “Euterpe” method with pre-recorded audio and video files (Bompard et al., 2023).

Art therapy for autism includes art-making activities (Datlen and Pandolfi, 2020). For ADHD, individual sessions have integrated relaxation activities, digital media exploration, and emotional release through expressive arts, as evidenced by a case study (Kim and Chung, 2024). Expressive arts therapy for older adults has involved visual art creation and storytelling (Luo et al., 2023). Dance movement therapy for autistic children has included mirroring, attunement, and structured improvisation (Moo and Ho, 2023).

### Prevalent delivery platforms and digital tools used in telehealth interventions of arts therapies for individuals with NNDs

Several different platforms and digital tools were employed; however, Zoom was the primary platform used to implement telehealth in the included studies (Clark et al., 2024; Dassa, 2024; Devlin, 2022; Kelly et al., 2024; Kim and Chung, 2024; Moo and Ho, 2023; Quigley and MacDonald, 2024; Tamplin et al., 2019; Tamplin et al., 2024). Specific sound settings, such as “Original sound for musicians” and “High-fidelity music mode,” were used to maintain music and voice quality by reducing automatic noise suppression (Tamplin et al., 2024). Zoom was also used to connect professionals with participants engaging in virtual platforms (Clark et al., 2024; Tamplin et al., 2019). Three music therapy studies used pre-recorded audio and video files on various devices (Bompard et al., 2023; Devlin, 2022; Stegemöller et al., 2020). Skype was used twice in music therapy studies (Baker and Krout, 2009; Quail et al., 2021). Live violin sessions were broadcasted using FaceTime (Bonakdarpour et al., 2021). WhatsApp was used in an art therapy study (Datlen and Pandolfi, 2020), and the Tencent conferencing tool was used for screen sharing in an expressive arts study (Luo et al., 2023).

### Benefits of telehealth arts therapies for individuals with NNDs

#### Research methods

Various qualitative and quantitative assessment methods were used in the included studies to evaluate the benefits of telehealth arts therapies for individuals with NNDs. Qualitative methods included structured and semi-structured interviews, therapist observations and reflections, and video elicitation techniques. In seven studies, therapists used structured and semi-structured interviews with participants or care persons to gain detailed insights into participants’ experiences and perceptions (Clark et al., 2024; Dassa, 2024; Kelly et al., 2024; Kim and Chung, 2024; Moo and Ho, 2023; Quigley and MacDonald, 2024; Tamplin et al., 2024). Observations and reflections of the therapist or care persons were reported in six studies as playing a significant role in the therapy process and benefits (Baker and Krout, 2009; Bonakdarpour et al., 2021; Dassa, 2024; Devlin, 2022; Quail et al., 2021; Datlen and Pandolfi, 2020; Kim and Chung, 2024). Video elicitation techniques were used in four studies (Baker and Krout, 2009; Quigley and MacDonald, 2024; Kim and Chung, 2024). Various quantitative research methods were used to measure psychological well-being, physiological health, and cognitive function. Measures included disease-specific scales and questionnaires (Dassa, 2024; Luo et al., 2023; Quail et al., 2021; Shah-Zamora et al., 2024; Tamplin et al., 2024), caregiver burden interviews (Shah-Zamora et al., 2024), voice measures (Stegemöller et al., 2020; Tamplin et al., 2024), usability and impact evaluations, and neuroimaging assessments (Luo et al., 2023).

#### Positive benefits

Each study included has demonstrated the positive benefits of telehealth arts therapies for individuals with NNDs. These include cognitive and emotional benefits, brain changes, and social benefits.

#### Cognitive and emotional benefits

In various studies, participants reported emotional enrichment and cognitive benefits. The specific nature of these benefits varies depending on the patient group and the type of method, but common themes include improved emotional state and cognitive function. For example, in individual cases involving two children, Devlin (2022) observed not only improved engagement and continued emotional and intellectual growth in a neurodivergent child but also sensory and emotional connectivity in a child with autism. Tamplin et al. (2024) and Shah-Zamora et al. (2024) noted emotional improvements in individuals with Parkinson’s disease following their respective therapeutic methods. While Tamplin et al. (2024) emphasized an increase in positive feelings, overall satisfaction, and a sense of being accepted and understood, Shah-Zamora et al. (2024) specifically noted a significant reduction in apathy and depressive symptoms.

However, there are differences in how these benefits manifest across different neurological disorders and methods. For example, Quail et al. (2021), in a case study of a patient with Alzheimer’s disease, focused on cognitive improvements, highlighting improvements in mood and cognition, along with increased engagement in meaningful activities, while Bonakdarpour et al. (2021) reported broader emotional benefits, including reduced stress and anxiety and overall high patient satisfaction among adults with various neurological disorders. Furthermore, Luo et al. (2023) found significant improvements in cognitive function for participants in the expressive arts group compared with a health education group. The study reported that the remote Expressive Arts Program positively impacted spontaneous brain activity and neural network connectivity. Specifically, there was increased activation in regions associated with memory and executive functions, including the right anterior cingulate/paracingulate cortex and the left dorsolateral superior frontal gyrus. Additionally, functional connectivity between the ventromedial prefrontal cortex and the left angular gyrus was enhanced, indicating strengthened brain network interactions. These neural alterations were linked to improved cognitive functioning and verbal abilities, supporting the feasibility of using internet technology to conduct proactive e-health interventions for patients with mild cognitive impairment (Luo et al., 2023).

#### Social benefits

The studies reviewed consistently highlighted significant social benefits, although the nature of these benefits varied by the target population and the therapy method. Common themes include enhanced social connectivity, improved relationships, and increased engagement. For example, Quigley and MacDonald (2024) found that participants with autism experienced increased social connectivity, greater accessibility, and convenience through the “Rock ‘n Roll Music Therapy Project” delivered via Zoom. This increase in social engagement is echoed in studies of individuals with dementia. Dassa (2024) observed that music therapy sessions rekindled couple relationships and helped spouses cope with daily challenges. Meanwhile, in two case examples, Kelly et al. (2024) noted reduced social isolation and increased self-confidence, improved relationships and communication. Studies of other neurological disorders and treatment modalities showed varying social benefits. Tamplin et al. (2019) found that virtual reality therapy reduced inhibitions about singing in front of others for individuals with spinal cord injury, highlighting the immersive and transportive nature of the therapy. Moo and Ho (2023) reported that dance movement therapy for autistic children with their parents resulted in enjoyment, improved understanding of the child, social development, and relationship building. Similarly, Luo et al. (2023) found that the expressive arts therapy group provided social support. Baker and Krout (2009) observed increased engagement, ability to work longer, and improved comfort and confidence in the case study of an individual with Asperger’s syndrome using Skype.

#### Mixed or no effect

Despite the generally positive results, some studies reported mixed or no significant effects in certain areas, often depending on the specific metrics assessed. For example, Tamplin et al. (2024) found that although participants with Parkinson’s disease experienced subjective emotional benefits, quantitative measures did not show significant improvements in voice measures or anxiety levels as assessed by the Depression Anxiety and Stress Scale. Similarly, in a study of a similar population, Shah-Zamora et al. (2024) found no significant improvements in quality of life, functional abilities, cognition, or caregiver burden after intervention. In other contexts, the effects were also mixed. Tamplin et al. (2019) noted that while virtual reality therapy reduced social inhibition in individuals with spinal cord injury, it may have reduced critical social cues. Likewise, Stegemöller et al. (2020) reported no significant differences in voice outcomes for people with Parkinson’s disease between pre-and post-intervention assessments, although they did observe improvements in respiratory control. Dassa (2024) acknowledged that despite the challenges and limitations of online therapy formats for people with dementia, the therapeutic settings were still beneficial, although not without issues. Key challenges highlighted in the study included technological difficulties, such as unstable internet connections and the lack of synchronization during music sessions, which disrupted the flow of interaction. Additionally, the emotional burden on caregiver spouses was a significant factor, as many found it challenging to maintain the musical practices independently once the sessions ended. Dassa (2024) study emphasized that while online music therapy had positive effects in rekindling relationships and alleviating some caregiving stress, the absence of the therapist’s guiding presence was a limitation to the long-term sustainability of the intervention.

## Discussion

This scoping review aimed to provide an in-depth overview of the current state of telehealth arts therapies for NNDs. It examined the types of disorders included, the interventions employed, the digital tools and technologies used, and the benefits of telehealth arts therapies. After an extensive literature search, 17 studies met the inclusion criteria and were thoroughly reviewed, covering various arts therapies such as music therapy, art therapy, expressive arts therapy, and dance movement therapy. Notably, no studies were identified on telehealth for drama therapy in this context, highlighting an immediate gap that warrants future exploration. The literature on telehealth arts therapies for NNDs dates back to 2009, with most studies appearing after 2020, coinciding with the COVID-19 pandemic. This period catalyzed the rapid adoption of telehealth, underscoring its potential benefits and feasibility (Doraiswamy et al., 2020). The substantial increase in studies after 2020 reflects the accelerated integration of telehealth into therapeutic practices.

The scoping review included studies on a range of NNDs, such as autism spectrum disorder, ADHD, dementia, and Parkinson’s disease, which are among the most common within these categories. Studies have shown that telehealth music therapy for people with Parkinson’s disease increases positive feelings, social connectivity, and vocal awareness (Tamplin et al., 2024). In cases of dementia, telehealth music therapy reinvigorated relationships between patients and their spouses and provided crucial emotional support (Dassa, 2024). In another example, therapy for older adults with mild cognitive impairment resulted in significant improvements and increased social and emotional support (Luo et al., 2023). Nonetheless, there is a notable absence of studies addressing rarer neurological conditions, such as neuroinfectious diseases, multiple sclerosis, and neuromuscular disorders, which may also benefit from telehealth arts therapy interventions.

Music therapy was the most studied of the telehealth arts therapies, although visual arts therapies have been noted for their more accessible adaptation to digital formats, possibly due to their lesser reliance on real-time interaction and synchronicity. It is essential to acknowledge that telehealth music therapy presents unique challenges, particularly regarding synchronicity and sound quality in sessions. Despite these hurdles, some music therapists have creatively navigated or circumvented these technological limitations. The predisposition of some music therapists to incorporate technology into their routine clinical practice might have facilitated a smoother transition to telehealth modalities. This adaptability may also be related to the therapist’s level of familiarity and comfort with technology, with those who were not averse to using technology finding the transition to telehealth more seamless. This focus on music therapy, while significant, indicates a disproportionate emphasis in the field, suggesting that other arts therapies, particularly visual arts, dance movement, and expressive therapies, remain underrepresented in telehealth research.

Furthermore, technological advances have allowed for high-quality audio transmission, which is essential for maintaining the therapeutic integrity of music sessions. This is evidenced by the adaptable sound settings of the most commonly used delivery tool, the Zoom platform, and specialized platforms for music therapy sessions (Clark et al., 2024). In the context of telehealth, art therapists have also incorporated virtual reality experiences and metaverse platforms (Tamplin et al., 2019; Kim and Chung, 2024), demonstrating the growing popularity of virtual reality in telehealth. These immersive experiences can increase patient engagement and therapeutic benefits (Lian, 2023).

The review identified diverse interventions and tools used in telehealth, demonstrating the versatility and adaptability of these modalities in remote settings and showing that the digital tools used in telehealth can enrich the therapeutic experience (Zubala et al., 2021). One of the recurring themes of the studies was the ability of online programs to overcome geographical barriers, enabling participation from remote and rural areas. These findings support the earlier statement that telehealth is vital for individuals facing mobility challenges or living in remote areas with limited healthcare resources (Cole et al., 2021).

All included studies highlighted numerous positive benefits of the telehealth of arts therapies, consistently reporting psychological enrichment, enhanced emotional state, social connectivity, and cognitive improvements. However, it is noteworthy that all 17 studies reported positive outcomes for telehealth arts therapies, which raises the possibility of a positive-results bias in the existing literature. This potential bias should be considered when interpreting these findings, as studies with less favorable outcomes may be underreported or unpublished. The methodological limitations identified in the reviewed studies frequently highlight challenges associated with small sample sizes, which limit the generalizability of findings. Many studies also lacked control groups or long-term follow-up evaluations, further constraining the ability to establish causal relationships and assess sustained effects of therapeutic interventions.

One crucial issue that has not been extensively addressed is the long-term sustainability and participation of patients in telehealth arts therapies. Maintaining patient motivation and adherence to good therapeutic practices over time can be challenging in remote settings where the physical presence of a professional is absent. Factors such as technological barriers, such as internet connectivity, and socioeconomic status could significantly influence the quality and accessibility of these therapies. Populations with lower technological literacy, limited internet access, or lower socioeconomic backgrounds may face significant barriers, limiting the reach and impact of telehealth in certain groups.

Another challenge is the need for standardized protocols and guidelines for telehealth arts therapies, which could lead to variability in the quality and benefits of these interventions. Addressing these issues through comprehensive longitudinal studies and developing standardized practices could increase the robustness and reliability of telehealth arts therapies, ensuring their broader applicability in managing NNDs.

### Recommendations

The findings of this scoping review suggest that telehealth arts therapies can address NNDs, particularly in scenarios where in-person sessions are impractical. However, this scoping review has highlighted the need for further research on all arts therapies, particularly drama therapy, where we could not find any research examples to include. Given the challenges associated with conducting randomized controlled trials in the creative arts therapies, future research should explore alternative methodologies to strengthen the evidence base. High-quality qualitative studies, such as phenomenological research and grounded theory, can provide deep insights into therapeutic processes and patient experiences. Detailed clinical reports and case studies also provide valuable contributions by contextualizing interventions and outcomes in real-world settings. Additionally, neuroimaging assessments are crucial to scientifically demonstrate the impact of telehealth interventions in art therapy for NNDs. Research on the long-term effects of telehealth therapies and their impact on caregiver burden and quality of life is also necessary.

### Limitations

This study has several limitations. The scoping review did not systematically evaluate the quality of the included studies, as scoping reviews aim to map the body of literature on a specific topic by identifying key themes and knowledge gaps. The articles reviewed and included were restricted to those written in English. Expanding the scope to include research published in other languages could improve the quantity and quality of the information obtained. We did not conduct a comprehensive backward or forward citation search, nor did we comprehensively screen the reference lists of all included studies. Therefore, it is possible that we missed some relevant studies because we did not review all abstracts or full-text articles from these reference lists. We acknowledge this as a limitation of our search strategy. Studies that did not involve qualified professionals were excluded. This exclusion criterion applies to studies where only students were present without the participation of a certified therapist, as well as to studies in which the therapy was conducted by a physical therapist, dance instructor, or nurse. The review focused on the experiences and therapy results of individuals with NNDs, thus excluding studies that described the experiences of therapists or staff. Including such studies in the research could broaden the scope and provide a more comprehensive understanding of the therapeutic process. Systematic reviews, book chapters, and gray literature were excluded from this review, which may have resulted in the omission of potentially valuable insights and data, as only primary research articles were included. It is possible that some articles were excluded at the abstract screening level if specific neurodevelopmental or neurological disorder terms were not explicitly mentioned in the title or abstract but only appeared in the full text. Despite a thorough literature review, the variability among the studies precluded the performance of meta-analyses. Consequently, the effect size and impact are limited due to this constraint. This scoping review was not formally registered, which is acknowledged as a potential limitation regarding the transparency and reproducibility of the process.

## Conclusion

Published articles on telehealth arts therapies for NNDs have integrated several findings, such as using expressive arts therapy programs can stimulate cognitive domains and promote neuroplasticity. These findings indicate that such therapies provide multisensory stimulation, regulate functional connectivity in the default mode network, and potentially improve cognitive function and emotional regulation. Specialists have conducted interventions in music therapy, art therapy, expressive arts therapy, and dance movement therapy. They have worked with various neurodevelopmental disorders, including autism spectrum disorders, ADHD, and neurological disorders such as stroke, epilepsy, cerebral palsy, CNS tumors, dementia, Alzheimer’s disease, Parkinson’s disease, spinal cord injuries, and mild cognitive impairment. Interventions used in telehealth arts therapies for individuals with NNDs are diverse, including therapeutic singing and songwriting, music listening, improvisation, musical recall exercises, group singing, relaxation, digital media exploration, art creation, and virtual reality experiences. The prevalent delivery platforms and tools used in telehealth interventions of arts therapies include Zoom, Skype, FaceTime, WhatsApp, Tencent, and specialized virtual environments alongside pre-recorded sessions. Telehealth arts therapies have demonstrated significant potential in improving the emotional, social, and cognitive well-being of individuals with NNDs. Arts therapies in telehealth settings are essential for individuals who are unable to attend face-to-face sessions for health or geographical reasons. As telehealth technologies continue to develop, arts therapies for individuals with NNDs delivered through these platforms are likely to become an increasingly important part of rehabilitation and support.

## Data Availability

The original contributions presented in the study are included in the article/Supplementary material, further inquiries can be directed to the corresponding author.

## Footnotes

^1^Professionals—art therapists, music therapists, dance movement therapists, expressive arts instructors.

^2^Telehealth- “use of electronic information and telecommunications technologies to support and promote long-distance clinical health care, patient and professional health-related education, and public health and health administration. Technologies include videoconferencing, the internet, store-and-forward imaging, streaming media, and landline and wireless communications” (U.S. Department of Health and Human Services, 2020). Telehealth encompasses four distinct applications: (a) live video (real-time, two-way interaction between a person and a provider using audiovisual telecommunications technology), (b) store-and-forward (photos, videos, and documents transmitted electronically), (c) remote patient monitoring (personal health and medical data collection from an individual in one location, which is transmitted to a provider in a different location for use in care and related support), (d) mobile health (health care and public health practice and education supported by mobile communication devices such as cell phones, tablet computers, and personal digital assistants) (Center for Connected Healthcare Policy, 2024).

^3^ www.rayyan.ai
