# Supplementary material for: Telehealth in arts therapies for neurodevelopmental and neurological disorders: a scoping review
Source: Front Psychol. 2024 Dec 18;15:1484726. doi: 10.3389/fpsyg.2024.1484726 (PMC11688619; doi:10.3389/fpsyg.2024.1484726)
Supplement: Supplementary file 1 [file Table_1.pdf]

Supplementary Material

Supplementary Table S1.

Main Data Extracted from the Included Studies in Music Therapy

Author(s)	Study design	Participants	Disorder	Therapy<br>1) time<br>2)intensity<br>3)duration	Therapeutic methods	Delivery platforms/ digital tools	Assessment tools	Results	Limitations
Baker and Krout (2009)<br>USA	Qualitative	15 year old boy	Asperger's Syndrome	1) Not exceeding 60 min<br>2) Once a week<br>3) For the face-to-face intervention song was completed in 1 session, for the Skype intervention in 2 sessions	(Individual)<br><br>Songwriting, using a 10-step song writing protocol, utilized in 2 settings:<br>1) Face-to-face<br>2) Via Skype	Skype	1) Video footage<br>2) Boy's written reflections<br>3) Music therapist's written reflections	Positive:<br>1) Increased engagement and ability to work longer, to finish the task<br>2) Increased ability for discussion and offering ideas from the boy’s part<br>3) Increased comfort (eye contact) and confidence	1) Single-case design<br>2) Controlled setting may not reflect true remote therapy<br>3) Technological issues impacted musical interaction
Bompard et al. (2023)<br>Italy	Quantitative	12 children under 12 completed the program	*n* = 7 genetic syndrome:<br>*n* = 1 a deletion on chromosome 13 (13q22.2)<br>*n* = 1 a deletion on chromosome 6 (6q16.1q16.2)<br>*n* = 1 a Rett syndrome	1) 5 min intervention (visual soundtrack)<br>2) 3 times a day<br>3) 12 days	(Individual with parent)<br><br>Home-based music therapy using the "Euterpe" method:<br>Audio:	Pre-recorded audio and video files demonstrated on any device (tablet, computer, etc)	1) Sleep Disturbance Scale for Children<br>2) Parent Stress Index-Short Form	Positive:<br>1) Improvement in sleep quality<br>2) Reduction in parental stress	1) Single-center study design<br>2) Lack of a control group<br>3) Small and heterogeneous sample size

			n = 1 a McKusick-Kaufman syndrome<br>*n* = 1 a partial duplication of 21q and of 7p with a karyotype 47 XX<br>*n* = 1 a mosaicism of chromosome 2<br>*n* = 1 a pluri-malformative syndrome<br><br>*n* = 5 cerebral palsy		1) Intro (40 sec): designed to capture and focus the child's attention<br>2) The maternal voice intertwined with the child's vocal reactions from previous sessions, layered over an effective piece of music from the child's personal audio archive<br>3) The most effective tracks previously administered during in-person therapies<br>4) "Lullaby song" sung by the mother to relax the child<br>Video:<br>Composed of a sequence of images from the child's previous therapy sessions				
Bonakdarpour et al. (2021)<br>USA	Mixed-methods	87 adults<br><br>post-session evaluation was completed by 65 patients	Stroke; epilepsy; central nervous system tumors; other neurological conditions	1) 30-40 min<br>2) Not mentioned<br>3) Lasted 3 months with 87 adults	(Individual)<br><br>1) Pre-intervention: participants selected their musical genres of interest and specified	FaceTime<br><br>Music practitioner: MacBook Air connected to Wi-Fi, using a Blue Yeti Nano	1) Observations<br>2) Post-session survey	Positive:<br>1) Intervention improved emotional state<br>2) Provided pleasurable experience<br>3) Reduced stress and anxiety	1) No pre/post-evaluation<br>2) Lack of a control group<br>3) Heterogeneous target population prevents specific conclusions about

					groups/bands they liked and identified music and groups they disliked<br><br>2) CDIM Live violin music, 5–8 pieces via FaceTime (CDIM: improvised music with pitches that are within the human vocal range at 131–524 Hz, slow tempi within 50–70 beats per minute, simple rhythms with no syncopation, interspersed with 10– 15s intervals of silence)	Professional Condenser USB Microphone<br><br>Patient: iPad		4) High Patient Satisfaction (98% would recommend)<br>5) Reduction in tension (blood pressure, heart rate, chest tightness, palpitations)<br>6) Increase in energy level	neurological conditions
Clark et al. (2024)<br>Australia	Qualitative	9 adults with care partner:<br><br>5 of them in spousal relationships<br><br>6 people with dementia and 5 care partners agreed to participate in post-program interviews	Dementia	1) 26-63 min<br>2) Not mentioned<br>3) 10 weeks	(Individual and group with care partner)<br><br>A two-phase approach:<br>1) Four duo sessions<br>2) Six group sessions, involving 2-4 duos<br><br>Online therapeutic	Zoom and Aeons platforms.<br><br>Equipment: (microphone, keyboard and guitar) and applications (Spotify, YouTube, GarageBand, Zoom whiteboard)	1) Semi-structured interviews followed by thematic analysis	Positive:<br>1) Positive impact on mood and emotional wellbeing<br>2) Social connection and collaboration, shared engagement<br>4) Positive impact on identity and life review<br>5) Cognitive stimulation	1) Text-only interview transcripts may not capture nuances in speech and non-verbal communication<br>2) The dual role of the facilitator in facilitating sessions, conducting interviews, and analyzing data

					songwriting program, including song parody, song collage, prose set to music, 'mini-opera' and original songwriting			6) Getting in touch with ones' creative and playful self	could introduce bias<br>3) Inclusion of both formal and informal care partners could introduce variability in the data
Dassa (2023) Israel	Qualitative	14 men (61-87 years) and their spouses	Dementia	1) 60 min<br>2) Once a week<br>3) 8 sessions, follow-up session 1 month later	(Individual with spouse)<br><br>1) Listening to music<br>2) Conversation about songs<br>3) Singing<br>4) Movement and dancing to familiar music or to the therapist's playing a musical instrument<br>5) Instruction how to use music beyond the therapy sessions	Zoom	1) Dementia Severity Rating Scale<br>2) Structured and semi-structured interviews<br>3) Music Therapy Session Reports<br>4) Clinical Supervision Reports	Positive:<br>1) The music therapy sessions rekindled the couple relationship<br>2) The therapist's presence helped the spouses cope with daily challenges<br><br>Mixed:<br>1) There were rather many challenges and limitations due to online format, but, despite of that, therapeutic setting can be provided and online music-therapy sessions can be beneficial	1) Findings are primarily based on reports from music therapists and caregiving spouses<br>2) Gender imbalance in the sample, with all participants being heterosexual couples where women were caregiving for male spouses<br>3) Limited preparation time for therapists to adapt techniques for online therapy due to the pandemic

Devlin (2022) USA	Qualitative	2 kids	Dexter: neurodivergentcy (elementary schooler)<br><br>Rafa: autism (4 years old)	1) 30 min<br>2) Dexter's Sessions: scheduled and carried out regularly<br>3) Dextrer-11 months, Rafa-15 months	(Individual)<br><br>Dexter: context of astronaut training; music listening; body movements; playing piano on "Shared Piano" app<br><br>Rafa: clear description of only the first session provided: welcome song; six songs from previous sessions, including a movement song and improvisational guitar play	Dexter's sessions: Zoom<br><br>Rafa's sessions: pre-recorded	1) Therapist observations and reflection	Positive:<br><br>Dexter:<br>1) Enhanced engagement and autonomy<br>2) Improved technological skills<br>3) Continued emotional and intellectual growth<br><br>Rafa:<br>1) Sensory and emotional connectivity<br>2) Familiarity and routine	Not mentioned
Kelly et al. (2024) Ireland	Qualitative	2 adults with their spouses	Dementia	1) 60 min<br>2) Once a week<br>3) 6 weeks	(Individual with spouse)<br><br>Program developed based on a three-tiered scaffold model devised by Knott and Block (2020):<br><br>1) Compilation of existing resources at Linktree page<br>2) Creation of original content (video recordings of participant's liked	Zoom	1) Online semi-structured interviews before/after interventions	Positive:<br>1) Reduced social isolation and improved confidence<br>2) Stimulation of reminiscence and happy memories<br>3) Enhanced relationship and communication<br>4) Increased accessibility through telehealth<br>5) Engagement and hope	1) Small sample size, limiting generalizability<br>2) Dual role of the authors as both researchers and facilitators, which may have influenced participant responses<br>3) Participants' pre-existing interest in music may have mediated positive outcomes, limiting applicability to non-musical populations

					songs; recordings of an original song composed during the sessions)<br>3) Synchronous telehealth music therapy sessions (receptive music; song singing; song discussion; songwriting)				
Quail et al. (2021)<br>UK	Mixed-methods	One adult in late 60s	Alzheimer's disease	Multimodal program:<br>1) 30 min<br>2) Twice a week, after 3 months three times per week<br>3) 4 months; then one month in-day centre person attendance; after that-3 months mixed online and day centre attendance	(Individual)<br><br>Online delivery program using:<br>1) Cognitive stimulation: picture recognition, puzzles, music quizzes, problem-solving, language skills and discussions on sport<br>2) Reminiscence therapy using pictures, photos and music<br>3) Music therapy including listening to pieces of music, playing an instrument and singing<br>4) Physical exercises including chair-based activities and ball games	1) Skype<br><br>2) YouTube videos (presented by the same music therapist)	1) Therapist and his wife observations+ patient's perspective<br>2) Number of assessment instruments:<br>a) Mini-Mental State Examination<br>b) Clinical Dementia Rating<br>c) Global Deterioration Scale<br>d) Functional Assessment Staging Test<br>e) Quality of Life in Alzheimer's Disease (patient, proxy)<br>f) Barthel Index<br>g) Neuro-psychiatric Inventory	Positive:<br>1) Improvement in mood and cognition<br>2) Engagement in Meaningful Activities<br>3) Reduction in Social Isolation<br>4) Continuity of Care	1) Lack of validated tools for remote dementia assessment limited formal outcome measurements<br>2) Reliance on qualitative observations instead of quantitative metrics<br>3) Technology barriers such as device availability and user familiarity impacted delivery and engagement<br>4) Safety-netting concerns limited the ability to detect environmental and symptom-related issues

					5) Reality orientation<br>6) Validation therapy		(severity, distress)		
Quigley and MacDonald (2024)<br>UK	Qualitative	2 adults<br><br>Participated in blended group with “healthy individuals”	Autism	1) 60 min<br>2) Twice a week<br>3) 8 weeks	(Group)<br><br>Rock'n Roll Music Therapy Project<br><br>Activities: musical and emotional recall exercises, improvisation, songwriting, group performance, process of recording two songs	Zoom	1) Semi-structured interviews<br>2) Video elicitation techniques (episodes with enjoyment; negative experience; when technology impacted quality of musical interaction)	Positive:<br>1) Psychological enrichment<br>2) Social connectivity<br>3) Creative expression<br>4) Increased accessibility and convenience	Not mentioned
Shah-Zamora et al. (2024)<br>USA	Mixed-methods	16 adults	Parkinson's disease	1) 60 min<br>2) Once a week<br>3) 12 weeks	(Small groups 5-6 pairs, patient and a caregiver)<br><br>1) Check-in (5-10 min)<br>2) Facial/Neck massage, oral movements, vocalizations (2 min)<br>3) Rhythmic warm-up/drumming (3-10 min)<br>4) Vocal exercises (10-15 min)<br>5) Therapeutic group singing (20-30 min)	A secure video-conferencing platform	1) Apathy Scale<br>2) Parkinson’s Disease Questionnarie<br>3) Schwab and England Activities of Daily Living<br>4) Beck Depression Inventory<br>5) Montreal Cognitive Assessment-Blind (MoCA-B)<br>6) Zarit Burden Interview<br>7) Multi-dimensional	Positive:<br>1) Significant reduction in apathy post-intervention compared to baseline with a large effect<br>2) A significant reduction in depressive symptoms post-intervention with a medium effect size<br><br>No effect:<br>1) No difference post-intervention in quality of life	1) Lack of a control group to compare outcomes<br>2) Small and homogeneous sample size, limiting generalizability<br>3) MoCA-B excluded executive function tasks, potentially limiting sensitivity to cognitive changes<br>4) Caregiver measures may not have been responsive to short-term changes

					6) Conversation/ Check-in (5-10 min)<br>7) Deep breathing (1-2 min)		Caregiver Strain Index<br>8) A Satisfaction Survey	2) No changes post-intervention in functional abilities or cognition<br>3) No impact on caregiver burden or strain	5) Self-administered surveys may have introduced response and social desirability bias
Stegemöller et al. (2020)<br>USA	Quantitative	8 adults	Parkinson's Disease	1) Not mentioned<br>2) Once a week<br>3) 8 weeks	(Group)<br><br>1) Group singing therapy program: series of vocal exercises; group singing of familiar songs<br><br>The intervention specifically targeted breath support, vocal intensity, fundamental frequency range	Pre- recorded therapeutic singing program	1) Voice and respiratory outcome measures collected 1 week before and 1 week after intervention<br><br>Voice outcome measures: phonation duration, phonation range, and vocal intensity<br><br>Respiratory outcome measures: maximal inspiratory pressure; maximum expiratory pressure	Positive:<br>1) Results are in keeping with results from a previous study using the exact same intervention with an in person therapist<br>2) For respiratory control, the mean for both outcome measures improved from pre-to post-assessment<br>3) Prerecorded group singing therapy program may be as effective as the in person for improving respiratory control in persons with Parkinson's Disease<br><br>Mixed:<br>1) No significant differences between the pre- and post-	1) Small sample size limits generalizability

								measures of the voice outcome measure	
Tamplin et al. (2024)<br>Australia	Mixed-methods	28 adults	Parkinson's disease	1) 90 min<br>2) Once a week<br>3) 12 weeks	(Group)<br><br>1) Breathing exercises and vocal warmups (approx. 15 min)<br>2) Speech exercises (approx. 10 min)<br>3) Intensive singing (approx. 35 min)<br>4) Social interaction practice (approx. 30 min)	Zoom<br><br>“Original sound for musicians” and “High-fidelity music mode”	1) Dysarthria Impact Scale<br>2) Depression Anxiety and Stress Scale<br>3) Lille Apathy Rating Scale-Short Form<br>4) The Parkinson’s Disease Questionnaire<br>5) Modified Movement Disorder Society-Unified Parkinson’s Disease Rating Scale<br>6) Survey before and after session<br>7) Speach loudness measurement and other voice outcomes	Positive:<br>1) Increased positive feelings and overall satisfaction<br>2) Increased vocal awareness of use of their voice<br>3) Social engagement<br>4) Feeling accepted and understood<br><br>No effect:<br>1) The quantitative measures did not show significant improvements in voice measures or anxiety measurements (by DASS) or energy levels	1) The sample included predominantly active singers.<br>2) The requirement for both initial and ongoing technological support posed challenges, with some participants needing continuous assistance to avoid session disruptions.<br>3) Therapists' limited experience with online platforms and the low digital literacy of some participants presented additional barriers to effective implementation

Tamplin et al. (2019)<br>Australia	Mixed-methods	6 adults<br><br>Phase 2 (feasibility and acceptability study)	Spinal cord injury	Single testing session	(Group)<br><br>1) Face-to-face session: participants sat together in a room with a music therapist and selected from a list of six well-known songs to sing<br><br>2) Zoom: therapist used the same song list and asked participants to sing together online with the music therapist<br><br>3) Participants entered Virtual reality via the app, selected their avatar, virtual location, and sang together with the music therapist in VR	Zoom video-conference with the JackTrip low-latency audio setup via Ethernet cabling.<br><br>MacBook Pro, Samsung phone and Gear VR headset, audio interface, headphones and cables.	1) System Usability Scale<br>2) Quebec User Evaluation of Satisfaction with assistive Technology<br>3) Psychosocial Impact of Assistive Devices Scale<br>4) An interview about experience	Positive:<br>1) Virtual reality as a positive experience<br>2) Virtual reality reduced inhibitions about singing in front of others<br>3) Virtual reality was immersive and transportative<br>4) Virtual reality equipment was comfortable, accessible and easy to use<br>5) A custom-designed virtual reality program, is acceptable and feasible mode of delivery for therapeutic singing interventions for people with spinal cord injury<br><br>Mixed:<br>1) Virtual reality may reduce social cues	1) Single testing session<br>2) Users could not view song lyrics while wearing VR headsets, limiting usability<br>3) High equipment cost (AUD 4000 per user), reducing clinical feasibility

Main Data Extracted from the Included Studies in Art Therapy

Author(s)	Study design	Participants	Disorder	Therapy 1)time 2)intensity 3)duration	Therapeutic methods	Delivery platforms/ tools	Assessment tools	Results	Limitations
Datlen and Pandolfi (2020) UK	Qualitative	5 young adults (23-27 years old)	Autism Spectrum Conditions (diagnoses included Cerebral Palsy, Scoliosis, Epilepsy, Sight and Hearing Impairments, and Global Delay Syndrome)	Not mentioned	(Group)<br><br>1) Art-making activities	WhatsApp	1) Feedback from members<br>2) Observations<br>2) Parents/carer observations	Positive:<br>1) Continued engagement and participation<br>2) Social inclusion and interaction	Not mentioned
Kim and Chung (2024) Republic of Korea	Qualitative	11-year-old boy	ADHD	1) Not mentioned.<br>2) Once a week.<br>3) 12 weeks	(Individual)<br><br>Each session systematically started with relaxation activities (making emotional emojis), including artwork activities, artwork discussions, and finishing<br><br><u>Sessions 1- 4:</u> Tension Reduction, Trust Building, exploration of Digital Media and Enjoyment of Art Activities<br><u>Sessions 5- 8:</u> Emotional Release Through Expressive Arts, Enhancement of	Zoom+ Metaverse platform ZEP<br><br>Pic collage app, Tooning.io, Emoji maker, Padlet, sketchpad app, AutoDraw app, Book Creator, MojiPop, Minecraft Education Edition app, Pixel Art Maker, Mozilla Hub	1) Researcher's observation records<br>2) Video recordings<br>3) Artworks<br>4) Three interviews with the participant, each 30 minutes – at the end of the initial stage, intermediate stage, and advanced stage of the study<br>5) 20-minute phone interviews with mother after each phase of art therapy sessions<br>6) Preliminary data before the study obtained from mother	Positive:<br>1) Enjoyable and self-directed art activities through gamification of metaverse<br>2) Enhanced presence and communication in the virtual environment<br>3) Expanded experience and immersion through convergence of virtuality and reality in metaverse<br>4) Emotional expression and emotional release through multisensory experiences in metaverse	1) Single-case study design limits generalizability.<br>2) Technical accessibility challenges due to the need for stable internet and appropriate hardware/software.<br>3) Lack of long-term outcome evaluation limits understanding of sustained effects

					Concentration through Creative Activation, Enhancement of Problem-Solving Skills<br>Sessions 9- 12:<br>Experience of Positive Emotions and Enhancement of<br>Achievement and Confidence			5) Personal growth and development through digital art therapy using metaverse	

Main Data Extracted from the Included Studies in Expressive Arts

Author(s)	Study design	Participants	Disorder	Therapy<br>1) time<br>2)intensity<br>3)duration	Therapeutic methods	Delivery platforms/ tools	Assessment tools	Results	Limitations
Luo et al.<br>(2023)<br>China	Quantitative (single-blind, two-arm parallel randomized controlled trial)	73 older adults divided into a remote Expressive Arts (EA) group<br>*n* = 38 and<br>*n* = 35 Health Education (HE) group	Mild cognitive impairment	1) 60 min<br>2) Twice a week<br>3) 12 weeks	(Group)<br>Two modules for experimental group: visual art creation and storytelling<br><br>The visual art: synchronous video teaching of art-themed activities and preset art-themed activity and related media preparation developed based on the Handbook of Art Therapy<br><br>Storytelling module: participant asked to create stories based on their visual artworks<br><br>Health Education group included 24 training courses on cognitive health	Sessions using an electronic device smartphone/ tablet/ computer<br><br>Tencent conference tool for sharing screen.	1) Montreal Cognitive Assessment<br>2) Mini-Mental State Examination<br>3) Auditory Verbal Learning Test<br>4) Verbal Fluency Test<br>5) Boston Naming Test<br>6) Symbol Digit Modalities Test<br>7) Lawton Instrumental Activities of Daily Living Scale<br>8) Neuro-imaging assessments: ReHo (Regional Homogeneity), Functional Connectivity (FC)	Positive:<br>1) The EA group-more significant improvements in cognitive function than the HE group<br>2) Affected spontaneous brain activity and brain networks<br>3) The ReHo values in the right anterior cingulate/ paracingulate cortex and the left dorsolateral superior frontal gyrus significantly increased<br>4) Decreased functional connectivity between the posterior cingulate cortex and the right middle temporal gyrus and increased functional connectivity between the ventromedial	1) Single-center study with a relatively small sample size<br>2) Baseline neuropsychological differences between groups may have exaggerated intervention effects<br>3) Time spent in contact with researchers was not quantified, limiting analysis of its impact<br>4) No inclusion of healthy controls, limiting functional connectivity comparisons<br>5) Short intervention duration may not capture long-term effects on brain function<br>6) Significant fMRI data attrition rate reduced the ability to detect therapeutic effects<br>7) Offline-only neuropsychological

								prefrontal cortex and left angular gyrus<br>5) Social and Emotional Support	assessment limited comprehensive evaluation of intervention effects

Main Data Extracted from the Included Studies in Dance Movement Therapy

Author(s)	Study design	Participants	Disorder	Therapy<br>1) time<br>2)intensity<br>3)duration	Intervention (steps, program)	Delivery platforms/ tools	Assessment tools	Results	Limitations
Moo and Ho (2023)<br>UK	Mixed-methods	5 families (parent-child dyads) signed up for the study<br><br>2 pairs completed the 6-week intervention	Autism	1) 45- 60 min<br>2) Once a week<br>3) 6 weeks	1) Meeting the child where they are at<br>2) Mirroring<br>3) Attunement<br>4) Improvisation<br>5) Developmental movement or Relationship Play<br><br>A consistent structure was used to ensure predictability. To address the child's difficulty with bodily cues via screen, the therapist adopted a directive approach, providing instructions, observing, and checking in with parents after each activity for insights	Zoom	1) Prior/after therapy: online individual, semi-structured interviews<br>2) Movement observation (recorded Zoom sessions)<br>3) Parent-Child Movement Scale<br>4) Laban Movement Analysis<br>5) Parent's feedback after each session	Positive:<br>1) Enjoyment<br>2) Improved understanding of child<br>3) Social development<br>4) Relationship-building<br>5) Insight and ideas<br>6) Tele-dance movement psychotherapy can be beneficial, perhaps particularly as an interim or adjunct form of therapy	1) Small sample size, limiting generalizability<br>2) Sample loss during the intervention, affecting inferences about its effects<br>3) Pandemic as a potential confounding factor impacting participants' behaviors and contexts<br>4) Participants not always visible in the camera frame, reducing opportunities for observation and intervention<br>5) Lack of prior in-person therapeutic relationships may have influenced engagement in teletherapy
